# Interferon-Induced Crohn’s Disease: An Unusual Side Effect of Interferon Therapy in a Patient With Chronic Hepatitis C Virus Infection

**DOI:** 10.7759/cureus.15568

**Published:** 2021-06-10

**Authors:** Omer Basar, Francis Dailey, Erica Dailey, Veysel Tahan, Ebubekir Daglilar

**Affiliations:** 1 Gastroenterology and Hepatology, University of Missouri-Columbia, Columbia, USA; 2 Gastroenterology and Hepatology, Kansas City Gastroenterology and Hepatology, Kansas City, USA

**Keywords:** interferon, crohn’s disease, inflammatory bowel diseases, hepatitis, adverse drug event

## Abstract

Gastrointestinal side effects of interferon (IFN) therapy for chronic hepatitis C virus (HCV) infection are non-specific. Rarely, this therapy has been reported to induce ischemic colitis and even ulcerative colitis. However, IFN-induced Crohn’s disease (CD) has previously been reported in only two individuals. We share our own experience of a patient treated for chronic HCV infection who developed CD after IFN therapy for chronic HCV infection.

A 28-year-old asymptomatic man with a history only of chronic HCV infection was treated with IFN and ribavirin, which he tolerated for 18 months and achieved sustained viral response (SVR). Halfway through the IFN regimen, he noticed infrequent painful bowel movements and bloody diarrhea. Following treatment, his symptoms resolved. Six months after therapy, colonoscopy showed a normal terminal ileum and colitis with skipped lesions and rectal sparing. Pathology demonstrated spotty chronic active colitis, with diffuse cryptitis, crypt distortion, and abundant abscesses, compatible with CD. The patient declined treatment and remained asymptomatic for two years. Labs including C-​reactive protein (CRP), erythrocyte sedimentation rate (ESR), fecal calprotectin, and celiac panel were normal. Upper GI endoscopy and capsule endoscopy were normal. Repeat colonoscopy showed normal terminal ileum and normal colonic mucosa, and biopsies of the terminal ileum and all segments of the colon were unremarkable. The patient was observed off treatment and has continued to remain asymptomatic, with a resolution of symptoms and disease continuing away from IFN exposure.

This is a rare case of CD induced by IFN, exhibiting significant importance regarding the evaluation of new cases of inflammatory bowel disease (IBD). Gastroenterologists need to keep in mind that INF therapy can be an uncommon cause of IBD.

## Introduction

The combination of interferon (IFN) and ribavirin was the treatment of choice for chronic hepatitis C virus (HCV) infection prior to the advent of direct-acting antiviral agents. Because IFN has only recently become phased out as the main therapy for chronic HCV infection, many individuals have a history of IFN exposure. This medication has an extensive side effect profile, including most commonly flu-like symptoms, bone marrow toxicity, elevated liver chemistries, fatigue, and psychiatric sequelae [1]. Gastrointestinal side effects of IFN tend to be non-specific and include anorexia, nausea, vomiting, diarrhea, and abdominal pain, among others [2]. Rarely, IFN has been reported to induce ischemic colitis [3], and even ulcerative colitis [4,5], in patients being treated for hepatitis C. However, to the best of our knowledge, IFN-induced Crohn’s disease (CD) has only been reported in the literature in two separate patients [6]. Here, we share our experience of a patient treated for chronic HCV infection who developed IFN-induced CD.

This work has been previously presented as an abstract (https://eventscribe.com/2018/ACG/ajaxcalls/PosterInfo.asp?PosterID=160376&efp=RFNSWFFHSFY2NDI0&rnd=0.294659).

## Case presentation

**Figure 1:**
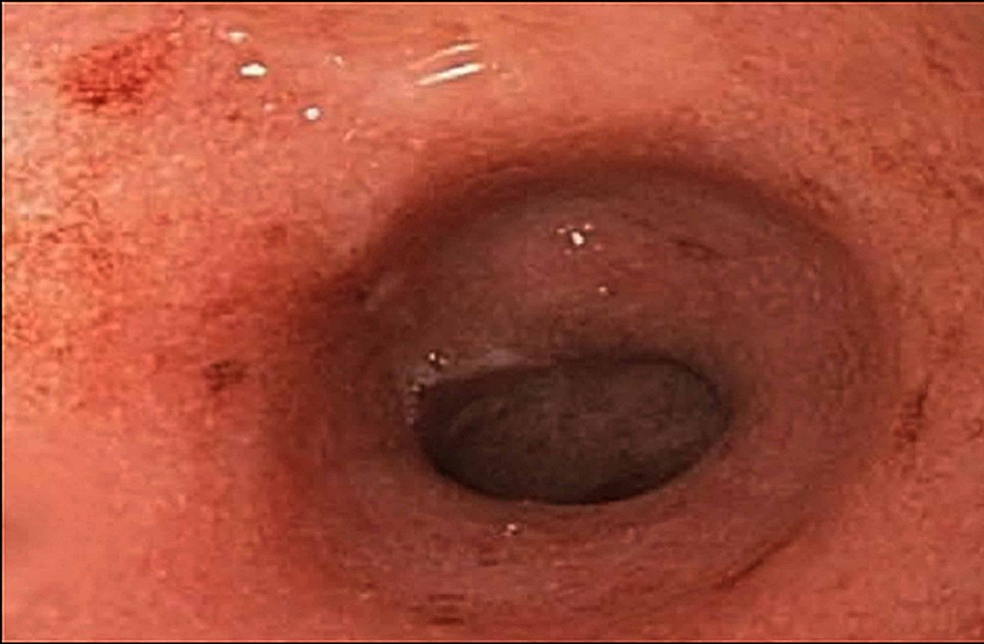
Initial colonoscopy Initial colonoscopy demonstrating colitis with skipped lesions and rectal sparing.

A 28-year-old man was referred to our clinic to establish care for CD. He was asymptomatic at the time of the visit and was not taking any medications. His past medical history was only significant for chronic HCV infection from remote intravenous drug abuse. He had no prior significant surgical history, family history, or social history. The patient was treated with IFN and ribavirin, which he tolerated for 18 months and achieved sustained viral response (SVR). Prior to this treatment period, the patient had no GI complaints. Halfway through the IFN therapy, the patient noticed infrequent painful bowel movements and occasional bloody diarrhea. Following completion of the IFN regimen, he felt better and his gastrointestinal complaints resolved. Six months after therapy, ileocolonoscopy showed a normal terminal ileum and colitis with skipped lesions and rectal sparing (Figure 1).

**Figure 2:**
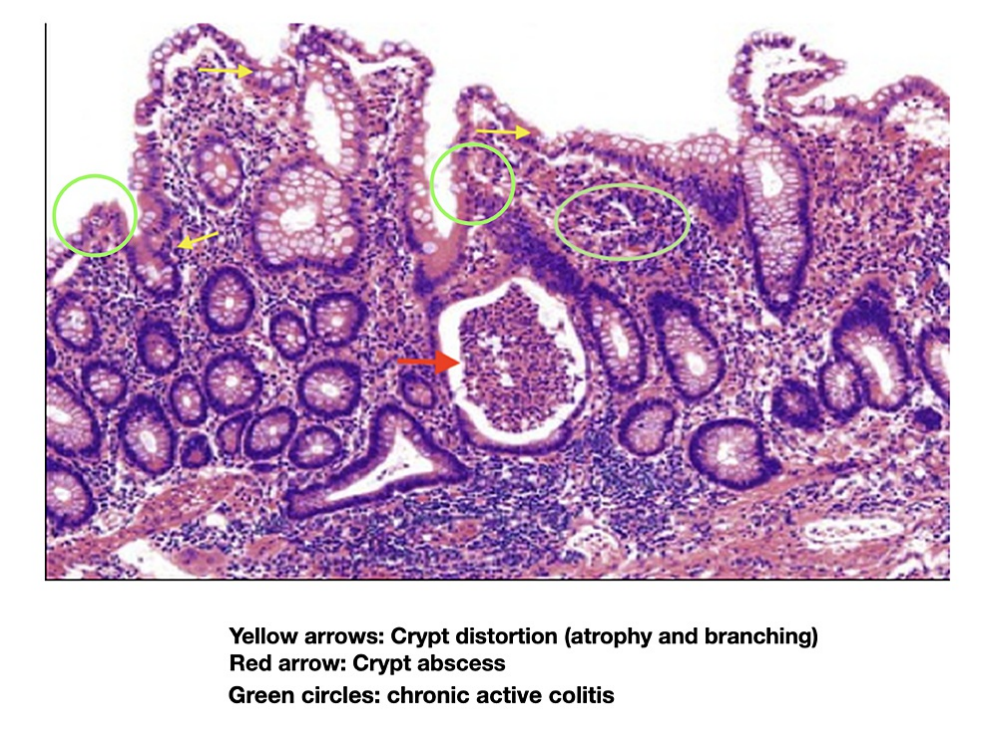
Histology Histology of biopsies from initial colonoscopy, demonstrating areas of chronic active colitis, with the presence of diffuse cryptitis, crypt distortion, and abundant abscesses, compatible with CD.

Pathology from segmental colon biopsies demonstrated spotty areas of chronic active colitis, with the presence of diffuse cryptitis, crypt distortion, and abundant abscesses, compatible with CD (Figure 2).

**Figure 3:**
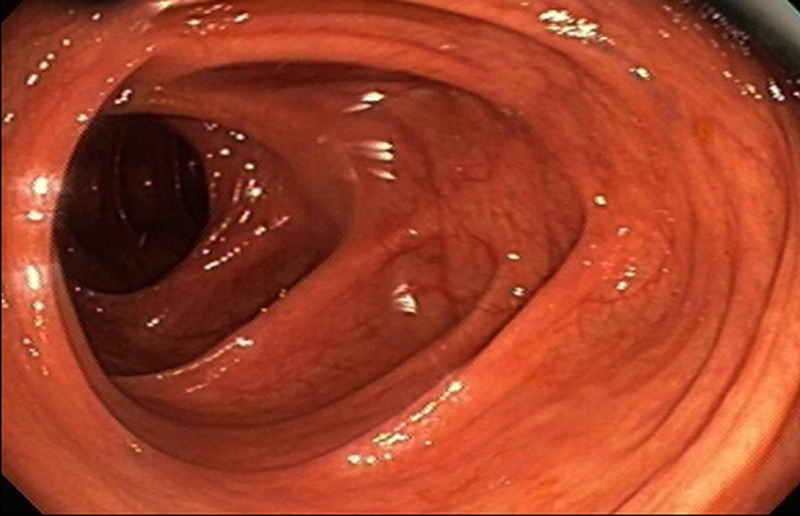
Repeat colonoscopy Follow-up colonoscopy, demonstrating normal colonic mucosa.

Since the patient was asymptomatic, he declined CD treatment. Over the next two years, he had occasional painless spotting with bowel movements. In our clinic, his vital signs and physical examination were completely unremarkable. Labs including complete blood count (CBC), comprehensive metabolic panel (CMP), C-​reactive protein (CRP), erythrocyte sedimentation rate (ESR), fecal calprotectin, and celiac panel were normal. Upper GI endoscopy and capsule endoscopy were normal, and repeat colonoscopy showed normal terminal ileum, normal colonic mucosa, and external, internal hemorrhoids (Figure 3).

Biopsies of all colon segments and the terminal ileum were unremarkable. 

Final diagnosis

The patient diagnosed as having IFN-induced CD as the cause of his previous signs and symptoms. The painless spotting was thought to be due to the hemorrhoids, as there were no other visible causes of hematochezia.

Treatment 

For the IFN-induced CD, the patient was actively observed off medication, given the symptomatic, endoscopic, and histologic resolution of CD. The hemorrhoids were treated with hydrocortisone cream for 14 days and the patient was placed on a high-fiber diet to have regular bowel movements.

Outcome and follow up

The patient has continued to remain asymptomatic over the last 24 months, with a resolution of symptoms and disease continuing away from the IFN exposure. He changed his diet to be higher in fruits and vegetables and is now having regular bowel movements that are soft, brown, and formed with no further evidence of spotting of blood.

## Discussion

IFN therapy consists of a large group of glycoproteins that can be used as an antiviral medication showing its effects on growth regulation, angiogenesis, cell differentiation, and immunomodulation. They are produced by leucocytes in response to viral infections and have been used to inhibit the replication of viruses, including HCV, hepatitis B virus (HBV), HIV, and others [7]. Many side effects have been previously reported, including fatigue, flu-like symptoms, depression, myelosuppression, thyroid disorders, and induction of autoimmune disorders. Gastrointestinal side effects of IFN are usually non-specific and include anorexia, nausea, vomiting, diarrhea, and abdominal pain. As previously mentioned, it has been rarely reported to cause ischemic and ulcerative colitis, and prior to this case, only two cases of CD have been reported due to IFN.

Ribavirin is a broad-spectrum nucleoside analogue with activity against a wide variety of RNA and DNA viruses, including influenza, RSV, HCV, HBV, and HIV. Even in the age of direct-acting antiviral agents for chronic HCV infection, ribavirin continues to have a role, including in difficult-to-treat populations such as patients with cirrhosis or resistance to oral antivirals [8]. The side-effect profile of ribavirin has been well documented, with anemia being the most common serious adverse effect, but others include neutropenia, thrombocytopenia, fatigue, flu-like symptoms, insomnia, pruritus, cough, headache, and other non-specific complaints [9].

Inflammatory bowel disease (IBD) is a unique spectrum of chronic disease processes, consisting primarily of CD and ulcerative colitis. While the exact pathophysiology remains unknown, it is characterized by chronic relapsing intestinal inflammation due to the interaction of genetic and environmental factors and immune-mediated involvement [10]. It is known that patients with IBD are at higher risk for HCV infection due to the need for endoscopy, surgery, and blood transfusions [11]. Consequently, IFN and ribavirin have been studied in the management of chronic HCV infection in those with IBD. Largely, this regimen has been found to be safe in this population, with only a few reports of worsening disease activity in those with ulcerative colitis [12,13]. Some clinical trials have even investigated the utility of IFN as a treatment for IBD given a decent safety profile and its possible positive effects on immunomodulation; unfortunately, while it was demonstrated to be safe, it was not found to be consistently effective in these studies [14,15].

Literature also showed retrospective data on the efficacy and safety of IFN and ribavirin therapy for chronic HCV infection in patients with IBD. In 2013, a large single-center retrospective review found a total of 15 individuals with concomitant IBD who underwent IFN-based treatment for HCV infection. Only one patient experienced an IBD exacerbation during therapy, and symptoms were controlled with mesalamine enemas. The rate of sustained virologic response was 67%. The authors concluded that in conjunction with published literature, the study findings demonstrated efficacy and safety of HCV infection therapy with IFN and ribavirin for patients with IBD similar to those without IBD [16]. Previous smaller case series and a cohort study have demonstrated similar findings, with IFN treatment for HCV infection showing some efficacy without affecting the course of CD [11,17-19]. Due to newer, oral direct-acting antiviral agents supplanting IFN and ribavirin as the treatment of choice for chronic HCV infection, further published work on IFN and ribavirin for HCV infection treatment in those with IBD will be unlikely to surface.

While ulcerative colitis due to IFN therapy has been reported several times in the literature, only two instances of CD due to IFN therapy have been published to the best of our knowledge. Similar to our case, Khalil et al. reported a young man with no major past medical history treated with IFN and ribavirin for chronic HCV infection. He achieved SVR within six months, but in the process, he developed weight loss and abdominal pain and rectorrhagia, with a drop in hemoglobin to 6.1 g/dL. Colonoscopy with biopsies demonstrated patchy ulcerations; biopsies revealed submucosal edema and digestive ulcerations without granuloma. He later developed pyoderma gangrenosum. The patient’s symptoms and anemia improved with prednisone then azathioprine and he achieved clinical remission. Khalil et al. also reported a middle-aged woman with a personal history of appendectomy and smoking, and a family history of CD in her sister, who was treated for chronic HCV cirrhosis with IFN and ribavirin for 48 weeks. She developed weight loss and abdominal pain and had an elevated CRP. Colonoscopy showed patchy deep cut-off ulcerations throughout the right colon and the ileocecal valve could not be traversed. Colon biopsies showed an inflammatory infiltrate with numerous neutrophils and eosinophils without granulomas. CT scan of the abdomen showed a severe stenosis of the last ileal loop. The patient was started on corticosteroids with the transition to azathioprine but did not improve, and ultimately needed TPN and ileocecectomy. Surgical pathology showed numerous granulomas throughout the entire specimen. The patient was started on long-term mesalamine and the CD was put into clinical remission [6].

Our patient and these previously reported cases of IFN-induced CD are supported by several features. In our case, the “Adverse Drug Reaction Probability Scale” developed in 1981 by Naranjo et al. was applied to assess the causality of IFN for CD [20]. This test was developed to standardize the assessment of causality for all adverse drug reactions and is widely available and utilized. In our case, the scale labels the association between IFN and CD as “probable.” While a positive rechallenge would change the label to “definitive,” this clearly would not be indicated given the SVR achieved and the significant side effect. In all three reported scenarios, the clinical presentation of symptoms during IFN therapy developed, with all patients experiencing abdominal pain and often also having hematochezia or rectorrhagia or malaise. In all cases, the endoscopic and histological findings supported the diagnosis of IBD, with only one case demonstrating granulomas on surgical pathology. Additionally, all three individuals achieved clinical remission further out from IFN exposure, with either no medication(s), with immunomodulators, or surgery plus mesalamine.

The underlying mechanisms by which IFN leads to the development of IBD in some individuals are difficult to determine and cannot be construed from these situations. Possibly, IFN may cause downstream immunological effects in genetically susceptible subjects leading to IBD. Only one of these three documented patients was at significant risk of CD with a positive family history, and she developed the most severe form of CD requiring surgery for ileal stenosis.

## Conclusions

Here, we presented an especially rare case of CD induced by IFN with ribavirin for the treatment of chronic HCV infection. While gastrointestinal side effects of IFN therapy are not uncommon, it has only infrequently been known to induce ischemic or ulcerative colitis in those treated for chronic HCV infection. In many case series and retrospective studies, IFN has been studied in individuals with CD and was not associated with significant side effects. Fortunately, our patient has had complete resolution of CD following active surveillance of medication. This case is important to share regarding the evaluation of new cases of IBD, particularly in the context of IFN for chronic HCV infection. It also suggests that special care should be exercised in the use of these drugs in patients with known IBD or with risk factors, including a positive family history for IBD. Gastroenterologists need to be aware of the uncommon causes of a common disorder as a CD.
